# SPARC silencing inhibits the growth of acute myeloid leukemia transformed from myelodysplastic syndrome via induction of cell cycle arrest and apoptosis

**DOI:** 10.3892/ijmm.2014.1648

**Published:** 2014-02-07

**Authors:** QING NIAN, QING XIAO, LI WANG, JING LUO, LI-PING CHEN, ZE-SONG YANG, LIN LIU

**Affiliations:** Department of Hematology, The First Affiliated Hospital of Chongqing Medical University, Chongqing 400016, P.R. China

**Keywords:** SPARC gene, myelodysplastic syndrome, transfection, the 5q- syndrome

## Abstract

Secreted protein acidic and rich in cysteine (SPARC) plays key roles in erythropoiesis; haploinsufficiency of SPARC is implicated in the progression of the 5q- syndrome. However, the role of SPARC in other subtypes of myelodysplastic syndrome (MDS) is not fully understood, particularly in the del(5q) type with a complex karyotype, which has a high risk to transform into acute myeloid leukemia (AML). In the present study, we investigated the role of SPARC in the proliferation and apoptosis of SKM-1 cells, an acute myeloid leukemia cell line transformed from an MDS cell line. SKM-1 cells were infected with SPARC-RNAi-LV or NC-GFP-LV lentivirus. Apoptosis and cell cycle profiling were assessed by flow cytometry, and cell proliferation was evaluated by MTS assay. The mRNA and protein expression levels of SPARC, p53, caspase-3, caspase-9 and Fas were detected by RT-PCR, real-time PCR and western blot assay. The SPARC shRNA constructed by us led to a significant reduction in SPARC expression in SKM-1 cells. SPARC knockdown inhibited the proliferation of SKM-1 cells by inducing cell cycle arrest at the G1/G0 phase and apoptosis. SPARC knockdown elevated the expression of p53, caspase-9, caspase-3 and Fas at both the mRNA and protein levels. SPARC silencing inhibited the growth of AML transformed from MDS by activating p53-induced apoptosis and cell cycle arrest. These data indicate that SPARC acts as an oncogene in transformed MDS/AML and is a potential therapeutic target in MDS/AML.

## Introduction

Myelodysplastic syndrome (MDS) encompasses a diverse group of neoplastic bone marrow disorders, characterized by abnormal cellular morphology and defects in the normal differentiation and proliferation of hematopoietic precursors. Various subtypes of MDS have a risk of transformation to acute myeloid leukemia (AML) (1,2). Recently, the chromosome aberrations in MDS have been elucidated; interstitial deletion of chromosome 5 is the most common cytogenetic abnormality in MDS (3). The del(5q) occurs either in isolation or together with another karyotypic abnormality (called complex karyotype). Isolated deletion of a segment of the long arm of chromosome 5q has been associated with a relatively favorable prognosis and a perceived low risk of progression to AML. In contrast, the complex karyotype has been associated with a poor prognosis and high risk of AML (4). In addition, a common deletion region (CDR) exists in the region of 5q31-32 (5). Research on the relationship between the CDR gene and cell biology can facilitate further understand of the pathogenesis of MDS/AML.

Secreted protein acidic and rich in cysteine (SPARC) has been described as a counter-adhesive, matricellular protein exhibiting a diversity of biological functions associated with morphogenesis, remodeling, cellular migration and proliferation (6). The expression of SPARC is varied in different types of cancers, and its role in tumorigenesis appears complex and is not well defined (7). For example, SPARC expression is high in breast and clorectal cancer (8,9), but low in prostate and lung cancer (10,11). In addition, haploinsufficiency of SPARC in the 5q- syndrome increases the adhesion of hematopoietic stem cells to supporting stromal cells and provides a clonal advantage (12). In 5q- syndrome, elevated SPARC expression inhibits the growth of tumor cells, while its low expression leads to tumor development. Therefore, lenalidomide has been used to treat patients with the 5q- syndrome via elevating SPARC expression (13).

MDS primarily affects the elderly and the prevalence is increasing; a majority of subtypes of MDS transform to AML (14). However, it is not clear whether SPARC plays a crucial role in MDS/AML. Therefore, exploring the function of genes in chromosome 5q, particularly in the CDR has important clinical value in transformed MDS/AML. Our study shed light on the pathogenesis of MDS/AML, and investigating the function of SPARC in transformed MDS/AML may provide new clues in the treatment and research of the transformation of MDS into AML.

In the present study, firstly we detected the expression of SPARC in SKM-1 cells. SKM-1 is a cell line derived from a male patient with AML transformed from MDS (15). We detected SPARC expression in SKM-1 cells by immunofluorescence. Then we employed lentiviral-mediated RNA interference to knock down SPARC in SKM-1 cells in order to determine its effects on the proliferation, apoptosis, and the expression of p53 and apoptosis-related genes in MDS/AML.

## Materials and methods

### Cell culture

The MDS/AML cell line SKM-1 was kindly provided by Professor Jianfeng Zhou at Tongji Medical College, Huazhong University of Science and Technology and was cultured in RPMI-1640 medium supplemented with 10% fetal bovine serum (Gibco BRL, Grand Island, NY, USA) in a humid atmosphere at 37°C with 5% CO_2_.

### Immunofluorescence

SKM-1 cells (10^6^ cells/ml) were incubated with polysorbate at 37°C for 1 h, washed with PBS three times and then incubated with 0.2% Triton for 1 h. Next the cells were incubated with the primary antibody (mouse anti-human SPARC monoclonal antibody; 1:1000; Abcam, USA) overnight, washed with PBS three times and incubated with a fluorescent secondary antibody (green fluorescent antibody; 1:1000; 2 h). After washing with PBS, the cells were observed under a fluorescence microscope.

### Construction of the SPARC recombinant lentivirus

A lentiviral vector containing a CMV-driven GFP reporter and a U6 promoter upstream of the cloning sites (*Age*I and *Eco*RI) was used for cloning small hairpin RNAs (shRNAs). The target sequence for SPARC was 5′-CCAGGTGGAAGTAGGAGAATT-3′, and the NC-GFP-LV sequence was 5′-TTCTCCGAACGTGTCACGT-3′. SPARC cDNA was amplified by reverse transcription-polymerase chain reaction (RT-PCR) and subcloned into a lentiviral vector RNAi-LV to construct a recombinant lentiviral vector named SPARC-RNAi-LV. According to the manual for packaging of the retrovirus in 293 cells, Lipofectamine 2000, pHelper 1.0 and pHelper 2.0 (Jikai Co., Shanghai, China) were used. The supernatants were then collected for determination of the viral titer.

### RNA interference

The cells were cultured in 6-well plates (10^6^ cells/ml) and infected with the lentivirus at a multiplicity of infection (MOI) of 100 for 10 h. The medium was replaced with basic medium. After 4 days, the cells were observed under a fluorescence microscope to evaluate the infection efficiency.

### RT-PCR

Total RNA of each group was extracted from the cells using RNAiso Plus (Takara Biotechnology, Dalian, China) and used for cDNA synthesis. Each well (25 μl reaction volume) contained 12.5 μl *Taq* (Takara Biotechnology), 1 μl of each primer (10 μmol/l), 2 μl cDNA template (50 ng/μl) and 8.5 μl ddH_2_O. PCR primers are listed in Table I. The cycling parameters were as follows: 97°C for 5 min, then 30 cycles of 97°C for 1 min, 56°C for 30 sec, and 72°C for 30 sec, and a final extension at 72°C for 7 min. All primers were designed using Primer 5 software and synthesized by Takara Biotechnology. RT-PCR results were analyzed using Quantity One software (Bio-Rad, Hercules, CA, USA).

### Real-time PCR

Quantitative real-time PCR was performed using an ABI PRISM 7500 real-time PCR system (Applied Biosystems, Foster City, CA, USA). The total reaction system was 25 μl: SYBR Premix Ex Taq II (12.5 μl), 1 μl of each primer (10 μmol/l) and 2 μl cDNA template (50 ng/μl), and ddH_2_O (8.5 μl). The primers were designed using Primer 5 software and synthesized by Takara Biotechnology, and are listed in Table II.

### Western blot analysis

Cells were lysed in 100 μl RIPA buffer supplemented with 1 μl PMSF, and the protein concentration of the lysate was determined using a BCA protein assay kit (Beyotime, Beijing, China). A total of 50 μg of protein per lane was separated by SDS-PAGE and transferred to PVDF membranes. The membranes were blocked with 5% skimmed milk for 2 h, and then incubated overnight at 4°C with the primary antibodies (mouse anti-human or rabbit anti-human monoclonal antibody; 1:1000; Abcam) for SPARC, p53, caspase-9, caspase-3 and Fas, followed by incubation with HRP-conjugated goat anti-rabbit or HRP-conjugated goat anti-mouse (1:1000) for 1 h at 37°C. Membranes were washed four times with TBST and developed using the ECL method. Band intensity was analyzed using Quantity One software.

### Cell proliferation assay

Cell proliferation was determined using an MTS assay. Cells were seeded at 500 cells/well into a 96-well plate. For the MTS assay, 20 μl of MTS (Promega, Madison, WI, USA) was added to each well and incubated at 37°C at 95% humidity and 5% CO_2_ for 1 h. The optical density at 490 nm was read with an enzyme immunoassay instrument (Bio-Rad).

### Annexin V and 7-AAD assay of apoptosis

Cells were collected (10^6^ cells/ml) and washed twice with PBS, suspended in 200 μl binding buffer, 1 μl Annexin V-PE and 5 μl 7-AAD (KeyGen Biotech, Shanghai, China) for 15 min in the dark. The apoptotic cells were determined by flow cytometry with CellQuest software (BD Biosciences, San Jose, CA, USA).

### Cell cycle distribution

Cells were collected and fixed with 70% anhydrous ethanol at 4°C overnight, and then incubated with RNase for 1 h at 37°C, followed by incubation with 100 μg/ml propidium iodide (PI) at room temperature for 30 min. The cell cycle profiles were analyzed using Multicycle software (USA).

### Statistical analysis

All results are expressed as means ± SE and were analyzed by GraphPad Prism 5 software. Each experiment was repeated three times. Comparison among groups was analyzed by one-way ANOVA. A P-value of <0.05 was considered to indicate a statistically significant result.

## Results

### Knockdown of SPARC by lentiviral-mediated RNAi in SKM-1 cells

**Figure 1:**
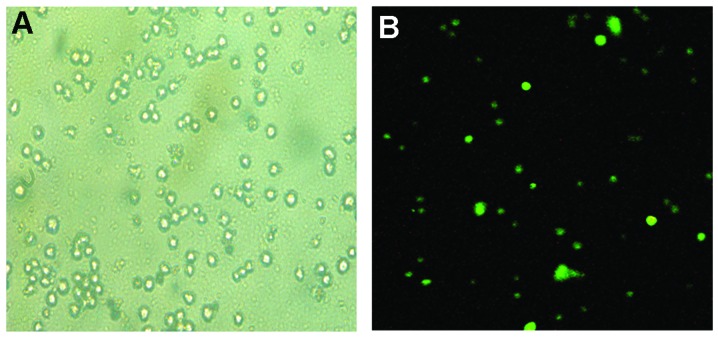
Expression of SPARC in SKM-1 cells. (A) SKM-1 cells. (B) SPARC expression in SKM-1 cells was detected by immunofluorescence (green fluorescence).

**Figure 2:**
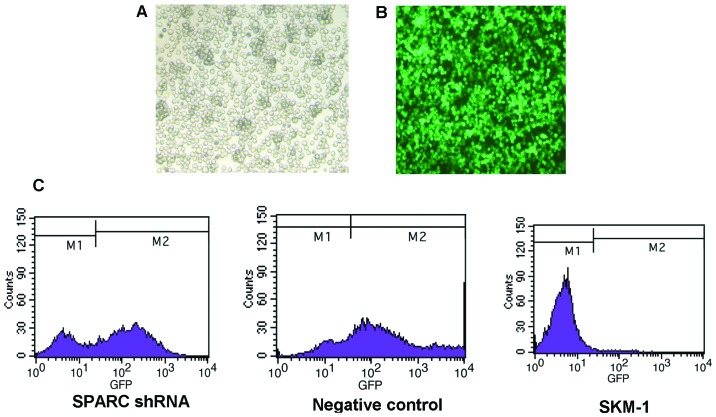
Infection of SKM-1 cells with SPARC shRNA. (A) Normal SKM-1 cells; (B) SKM-1 cells infected with SPARC shRNA. (C) Transfection efficiency was detected by flow cytometry. M1, no EGFP; M2, EGFP. The transfection efficiency of SPARC shRNA was 67.88%, the negative control was 79.88%, and SKM-1 was 0.2%.

**Figure 3:**
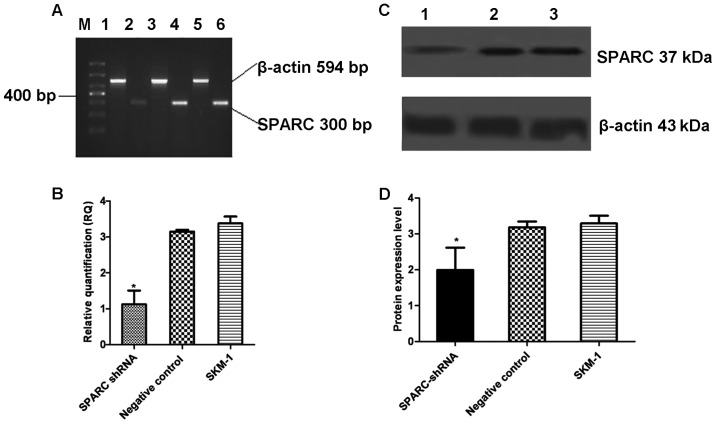
SPARC knockdown in SKM-1 cells. (A) RT-PCR showing the expression of SPARC mRNA. Lanes 1, 3 and 5, human actin; lane 2, SPARC shRNA; lane 4, negative control; lane 6, SKM-1. (B) Real-time PCR showing the relative expression of SPARC mRNA in the different groups. ^*^P<0.05 vs. other groups. (C) Western blot analysis showing the expression of SPARC protein. Lane 1, SPARC shRNA-infected cells; lane 2, negative group; lane 3, SKM-1. (D) Protein expression levels in the different groups.

The immunofluorescence analysis demonstrated that SPARC was abundantly expressed in the SKM-1 cells (Fig. 1). Next we constructed the SPARC shRNA lentiviral vector and infected the SKM-1 cells with SPARC shRNA. Following infection, we found that >60% of cells were GFP-positive, indicating high infection efficiency (Fig. 2). RT-PCR, real-time PCR and western blot analysis showed that SPARC shRNA significantly reduced the expression of SPARC at both the mRNA and protein levels (Fig. 3).

### Knockdown of SPARC inhibits MDS/AML cell proliferation

**Figure 4:**
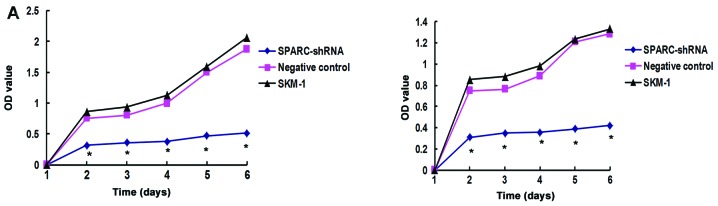

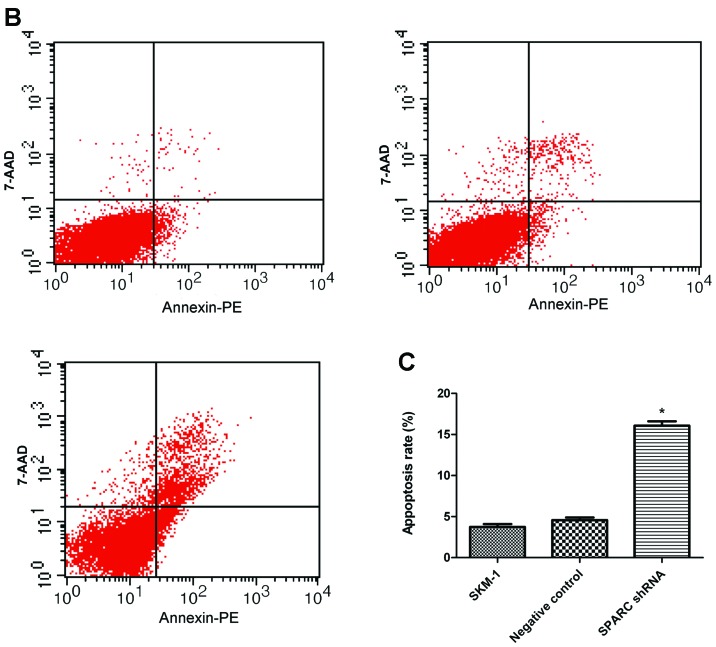

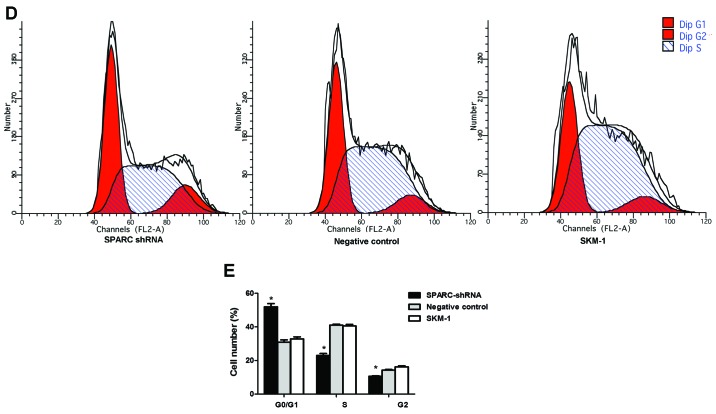
Effects of SPARC knockdown on proliferation, cell cycle and apoptosis of SKM-1 cells. (A) Cell proliferation was examined by MTS assay. ^*^P<0.05. (B) Apoptosis was determined by Annexin V assay. (C) Quantitative analysis of the apoptotic rate, ^*^P<0.05. (D) Cell cycle was examined by flow cytometry. (E) Quantitative analysis of the cell numbers (%) in the different phases, ^*^P<0.05 vs. other groups. All data were analyzed by GraphPad Prism 5 software.

To evaluate the effects of SPARC knockdown on the proliferation of SKM-1 cells, we performed an MTS assay. After 2 days, the OD value of cells treated with SPARC shRNA decreased to 0.5–0.7 when compared to the OD values in the negative control and SKM-1 cells. The results showed that knockdown of SPARC inhibited the proliferation of SKM-1 cells (Fig. 4A).

### Knockdown of SPARC induces cell cycle arrest at the G1/G0 phase and apoptosis in MDS/AML cells

To elucidate the mechanism by which knockdown of SPARC inhibits the proliferation of MDS/AML cells, we performed flow cytometric analysis to evaluate the cell cycle. As shown in Fig. 4D, 40–50% of SKM-1 cells infected with SPARC shRNA were arrested at the G1 or G1/G0 phase (P<0.05) compared with 10–20% in the negative control and SKM-1 cells (Fig. 4E). In addition, we performed flow cytometry analysis to evaluate apoptosis and found that ~20% cells were induced to apoptosis after infection with SPARC shRNA. The percentage of apoptotic cells was much higher in the SPARC shRNA-infected cells than these percentages in the other two groups (P<0.05) (Fig. 4B and C). Taken together, these data indicate that knockdown of SPARC inhibits the proliferation of MDS/AML cells by inducing cell cycle arrest and apoptosis.

### Knockdown of SPARC leads to upregulation of expression of apoptotic factors in MDS/AML cells

**Figure 5:**
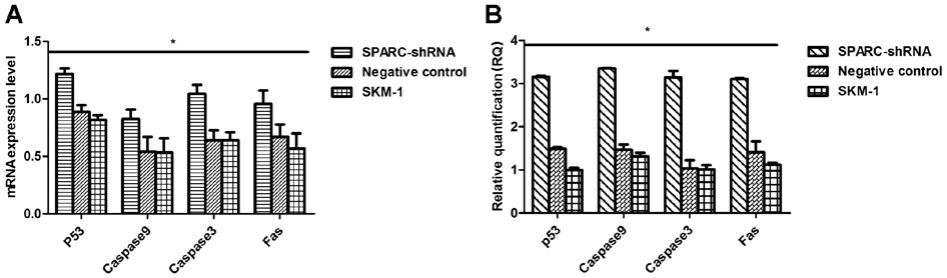

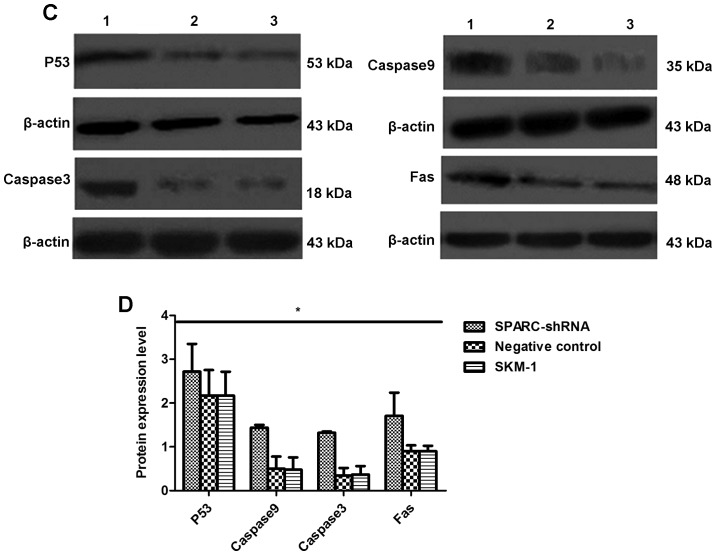
Effects of SPARC on the expression of apoptosis-related proteins in SKM-1 cells. (A) RT-PCR analysis of the mRNA expression of the indicated genes p53, caspase-9, capase-3 and Fas. Quantitative analysis of RT-PCR results by Quantity One software. ^*^P<0.05, SPARC shRNA group vs. other two groups. (B) Real-time PCR analysis of the mRNA expression of the indicated genes in the different groups. ^*^P<0.05, SPARC shRNA group vs. other groups. (C) Western blot analysis of the expression of the indicated proteins in the different groups. Lane 1, SPARC shRNA-infected group; lane 2 negative group; lane 3, SKM-1. (D) Quantitative analysis of the western blot results by Quantity One software ^*^P<0.05, SPARC shRNA group vs. the other groups.

To understand the molecular mechanism by which SPARC regulates apoptosis, we examined the expression of apoptotic factors such as p53, caspase-3, caspase-9 and Fas in MDS/AML cells with SPARC knockdown. RT-PCR, real-time PCR and western blot analysis showed that the expression of p53, caspase-3, caspase-9 and Fas at both the mRNA and protein levels was obviously higher in the SPARC shRNA-infected cells than levels in the other two groups (Fig. 5). These results suggest that SPARC inhibits MDS/AML cell apoptosis by downregulating the expression of p53, caspase-3, caspase-9 and Fas.

## Discussion

In the present study, we aimed to investigate the role of SPARC in the transformation of MDS into AML. Thus we selected a human MDS/AML cell line, SKM-1, with complex abnormal karyotype including del(9q), i(17q) and t(17p) (16,17). Following knockdown of SPARC, a del(5q)-like model which is an ideal model for this study was obtained. First, abundant expression of SPARC in SKM-1 cells was confirmed by immunofluorescence. Then we used lentiviral-mediated shRNA to knockdown the expression of SPARC in SKM-1 cells.

In order to better understand the function of SPARC in SKM-1 cells, we analyzed cell proliferation and apoptosis. By MTS assay, knockdown of SPARC suppressed MDS/AML cell proliferation. Flow cytometric analysis indicated that knockdown of SPARC increased the number of cells in the G1/G0 phase, while decreasing the number of cells in the S phase, revealing G1/G0 phase arrest. In addition, we found increased apoptosis in the MDS/AML cells after knockdown of SPARC. SPARC is a potential tumor-suppressor gene in 5q- syndrome, and is thought to have anti-adhesive effects, thereby promoting apoptosis. It also regulates cellular migration and proliferation. Based on the results of the MTS assay and flow cytometry in our study, decreased expression of SPARC pointed to a contrary conclusion. SPARC was found to be differentially expressed in tumors and its surrounding stroma in various types of cancers in comparison to normal tissue. To understand the mechanism of SPARC in the proliferation and apoptosis of transformed MDS/AML, we detected the expression of p53, caspase-3, caspase-9 and Fas in the SPARC shRNA-infected cells and in the other groups.

Recently, expression of SPARC was correlated with p53 in MDS. The haploinsufficiency of SPARC induced the increased expression of p53 in the 5q- syndrome (18). p53 is a well-known tumor suppressor that impedes the cell cycle at the G1-S checkpoint (19). To understand the association between p53 and SPARC which regulates the proliferation and apoptosis of MDS/AML cells, we detected the expression of p53 in SPARC shRNA-infected cells, negative control cells and SKM-1 cells. We found that knockdown of SPARC increased the expression of p53. Thus, we believe that cell growth regulation by SPARC is related to the expression of p53 in MDS/AML. These results are generally in agreement with previous studies concerning ovarian cancer (20), glioma (21), gastric cancer (22) and melanoma cells (23) and the relationship of p53.

Furthermore, since SPARC knockdown induced cell apoptosis and reduced proliferation, while p53 expression was increased, we assumed that the intrinsic pathway of apoptosis should be regulated by apoptosis-related genes. Therefore, we detected the expression of caspase-9, caspase-3 and Fas which are common apoptosis-related genes. Upon apoptosome formation, caspase-9 becomes catalytically active and acts on downstream target caspase-3 (24,25). In the present study, we found that knockdown of SPARC increased the expression of caspase-9 and caspase-3. These results suggest that knockdown of SPARC activates caspase-9 and the downstream molecule, caspase-3. In addition, Fas is an upstream factor in the apoptosis pathway (26). We found that its expression was increased in SKM-1 cells following SPARC knockdown. Programmed cell death, apoptosis, is vital for normal development and cell homeostasis. In brief, SPARC plays an important role in MDS/AML cell proliferation by controlling cell-related apoptosis genes, but whether these signaling pathway genes have a relationship with transformed MDS/AML, and whether these increases are contributing factors to the transformation of MDS into AML require further study. These results suggest that the regulation of the expression of these apoptosis-related proteins may be used to prevent the transformation of MDS to AML and cell proliferation.

In conclusion, we present evidence that SPARC regulates SKM-1 cell proliferation and apoptosis, which is associated with the expression of p53. Knockdown of SPARC inhibits cell proliferation, induces cell cycle arrest and apoptosis and activates the p53 pathway. These data suggest that SPARC may act as an oncogene in transformed MDS/AML while it acts as a tumor-suppressor in 5q- syndrome. In combination with the results of our previous study (27), 5q- syndrome has a favorable prognosis and this may be related to the relative genetic stability as evidenced by an absence of other cytogenetic abnormalities and a failure to find other leukemia-associated mutations in the 5q- syndrome group in contrast to other MDS patients with the del(5q). Thus, we conclude that high-risk MDS with del(5q) may possess a totally different pathophysiology with the 5q- syndrome which may explain the reason for the transformation to AML resulting in poor prognosis. These findings may be of pathogenetic importance in the MDS transformation into AML and suggest that SPARC is a potential therapeutic target for MDS/AML.

## Figures and Tables

**Table I:** RT-PCR primers.

Genes	Forward and reverse primers	Product length (bp)
SPARC	F: 5′-ACCTGTCACTGTCTTGTACCCTTGT-3′ R: 5′-CGGCGTTTGGAGTGGTAGAA-3′	300
Actin	F: 5′-GTGATCTTGGACTTGATATTGGTG-3′ R: 5′-GTCCATACCCAAGGCATCCTG-3′	594
P53	F: 5′-CCTCCTCAGCATCTTATCCGA-3′ R: 5′-GTGCTCGCTTAGTGCTCCCT-3′	312
Caspase-3	F: 5′-GGTTCTGGAGGATTTGGTGATG-3′ R: 5′-GACGCCGCAACTTCTCACAG-3′	320
Caspase-9	F: 5′-TGTGGTGGGGAGCAGAAAGA-3′ R: 5′-TTCACCGAAACAGCATTAGCG-3′	323
Fas	F: 5′-CAATTCTGCCATAAGCCCTGT-3′ R: 5′-CTTGGTGTTGCTGGTGAGTGT-3′	324

**Table II:** Real-time PCR primers used in this study.

Genes	Forward and reverse primers	Product length (bp)
SPARC	F: 5′-GGCCTGGATCTTTCTCCTT-3′ R: 5′-CCCACAGATACCTCACCTC-3′	126
Actin	F: 5′-CCACGAAACTACCTTCAACTAA-3′ R: 5′-GTGATCTCCTTCTGCATCCTGT-3′	132
P53	F: 5′-CTTTGAGGTGCGTGTTTGTG-3′ R: 5′-GTTGGGCAGTGCTCGCTTAG-3′	124
Caspase-3	F: 5′-GGCATTGAGACAGACAGTGGTG-3′ R: 5′-GGCACAAAGCGACTGGATGA-3′	153
Caspase-9	F: 5′-GGTTCTGGAGGATTTGGTGATG-3′ R: 5′-GACGCCGCAACTTCTCACAG-3′	184
Fas	F: 5′-ATCTGGACCCTCCTACCTCTGG-3′ R: 5′-GATGCAGGCCTTCCAAGTTCT-3′	151

## References

[1] Nimer SD (2008). Myelodysplastic syndromes. Blood.

[2] Ghariani I, Braham N, Hassine M, Kortas M (2013). Myelodysplastic syndrome classification. Ann Biol Clin.

[3] Jädersten M, Hellström-Lindberg E (2010). New clues to the molecular pathogenesis of myelodysplastic syndromes. Exp Cell Res.

[4] Davids MS, Steensma DP (2010). The molecular pathogenesis of myelodysplastic syndromes. Cancer Biol Ther.

[5] Giagounidis AA, Germing U, Wainscoat JS (2004). The 5q- syndrome. Hematology.

[6] Swaminathan SS, Oh DJ, Kang MH (2013). Secreted protein acidic and rich in cysteine (SPARC)-null mice exhibit more uniform outflow. Invest Ophthalmol Vis Sci.

[7] Rotllant J, Liu D, Yan YL (2008). Sparc (osteonectin) functions in morphogenesis of the pharyngeal skeleton and inner ear. Matrix Biol.

[8] Hsiao YH, Lien HC, Hwa HL (2010). SPARC (osteonectin) in breast tumors of different histologic types and its role in the outcome of invasive ductal carcinoma. Breast J.

[9] Wiese AH, Auer J, Lassmann S (2007). Identification of gene signatures for invasive colorectal tumor cells. Cancer Detect Prev.

[10] Said N, Frierson HF, Chernauskas D (2009). The role of SPARC in the TRAMP model of prostate carcinogenesis and progression. Oncogene.

[11] Isler SG, Ludwig CU, Chiquet-Ehrismann R (2004). Evidence for transcriptional repression of SPARC-like 1, a gene downregulated in human lung tumors. Int J Oncol.

[12] Tohyama K (2012). 5q- syndrome, MDS with isolated del(5q). Nihon Rinsho.

[13] Duong VH, Komrokji RS, List AF (2012). Efficacy and safety of lenalidomide in patients with myelodysplastic syndrome with chromosome 5q deletion. Ther Adv Hematol.

[14] Usmani SZ, Sawyer J, Rosenthal A (2013). Risk factors for MDS and acute leukemia following total therapy 2 and 3 for multiple myeloma. Blood.

[15] Nakagawa T, Matozaki S, Murayama T (1993). Establishment of a leukaemic cell line from a patient with acquisition of chromosomal abnormalities during disease progression in myelodysplastic syndrome. Br J Haematol.

[16] Nakagawa T, Matozaki S (1995). The SKM-1 leukemic cell line established from a patient with progression to myelomonocytic leukemia in myelodysplastic syndrome (MDS) - contribution to better understanding of MDS. Leuk Lymphoma.

[17] Kimura S, Kuramoto K, Homan J (2012). Antiproliferative and antitumor effects of azacitidine against the human myelodysplastic syndrome cell line SKM-1. Anticancer Res.

[18] Barlow JL, Drynan LF, Hewett DR (2010). A p53-dependent mechanism underlies macrocytic anemia in a mouse model of human 5q- syndrome. Nat Med.

[19] Liebermann DA, Hoffman B, Vesely D (2007). p53 induced growth arrest versus apoptosis and its modulation by survival cytokines. Cell Cycle.

[20] Yiu GK, Chan WY, Ng SW (2001). SPARC (secreted protein acidic and rich in cysteine) induces apoptosis in ovarian cancer cells. Am J Pathol.

[21] Seno T, Harada H, Kohno S (2009). Downregulation of SPARC expression inhibits cell migration and invasion in malignant gliomas. Int J Oncol.

[22] Yin J, Chen G, Liu Y (2010). Downregulation of SPARC expression decreases gastric cancer cellular invasion and survival. J Exp Clin Cancer Res.

[23] Horie K, Tsuchihara M, Nakatsura T (2010). Silencing of secreted protein acidic and rich in cysteine inhibits the growth of human melanoma cells with G arrest induction. Cancer Sci.

[24] Khalil H, Peltzer N, Walicki J (2012). Caspase-3 protects stressed organs against cell death. Mol Cell Biol.

[25] Brentnall M, Rodriguez-Menocal L, De Guevara RL (2013). Caspase-9, caspase-3 and caspase-7 have distinct roles during intrinsic apoptosis. BMC Cell Biol.

[26] Li Q, Wang Y, Wang Y (2013). Distinct different sensitivity of Treg and Th17 cells to Fas-mediated apoptosis signaling in patients with acute coronary syndrome. Int J Clin Exp Pathol.

[27] Wang L, Fidler C, Nadig N (2008). Genome-wide analysis of copy number changes and loss of heterozygosity in myelodysplastic syndrome with del(5q) using high-density single nucleotide polymorphism arrays. Haematologica.

